# The Case for Maternal Postpartum Deworming

**DOI:** 10.1371/journal.pntd.0005203

**Published:** 2017-01-05

**Authors:** Layla S. Mofid, Theresa W. Gyorkos

**Affiliations:** 1 Department of Epidemiology, Biostatistics and Occupational Health, McGill University, Montréal, Québec, Canada; 2 Division of Clinical Epidemiology, Research Institute of the McGill University Health Centre, Montréal, Québec, Canada; University of Queensland, AUSTRALIA

## Worms and Women

Infection with *Ascaris lumbricoides* (roundworm), *Trichuris trichiura* (whipworm), and *Necator americanus* and *Ancylostoma duodenale* (hookworms)—collectively referred to as the soil-transmitted helminths (STHs)—are among the most prevalent infections of humankind, co-occurring on all inhabited continents [1]. Those most vulnerable, who reside in endemic areas where adverse health, social, and economic conditions predominate, carry the greatest STH-attributable disease burden [2]. STHs contribute to malnutrition through blood loss, iron deficiency anemia, and nutrient malabsorption [3]. STHs have been linked to impaired growth, cognitive deficits, lower educational achievement, and reduced economic productivity in adulthood [4], thus perpetuating the cycle of poverty into future generations.

Periodic preventive chemotherapy (PC) with anthelminthic drugs (i.e., deworming) is the cornerstone of prevention and control measures against STH infections in highly endemic areas [2]. The World Health Organization (WHO) has recommended that PC programs target the three groups at highest risk of infection: school-aged children (SAC), preschool-aged children (pre-SAC), and women of reproductive age (WRA). The WHO Preventive Chemotherapy Databank reports on deworming coverage for SAC and pre-SAC; however, no coverage estimates are available for deworming in WRA, and few countries include this risk group among deworming activities. In fact, the disease burden of STHs in WRA is largely unknown. In sub-Saharan Africa alone, it has been estimated that almost 40 million WRA are infected with hookworm, including more than a quarter of pregnant women (26.7%) [5]. Here, we discuss the need for including WRA in large-scale national deworming programs as a means of improving their own health, the health of their children, and as a strategy to complement global strategic plans for STH control.

## Current Deworming Strategies and 2020 Targets

In 2012, WHO proposed a strategic plan to eliminate STHs as a public health problem in children by 2020 [2]. Specific targets include at least 75% coverage of PC in SAC and pre-SAC living in endemic areas. However, recent predictive modelling suggests that current PC efforts targeting children may not be sufficient to meet the 2020 goal of controlling STHs without improving coverage and including WRA, particularly in regions where hookworm infection predominates [6].

## WRA: A High-Risk Group Often Forgotten

In many countries, WRA suffer from high levels of anemia, in part due to their underlying poor iron status, increased iron demands, and blood loss due to menstruation, pregnancy, and lactation [7]. Such realities make WRA particularly vulnerable to the detrimental effects of STH infections caused by intestinal blood loss and malabsorption of micronutrients [2]. Of particular importance are infections with both hookworm and *T*. *trichiura*, owing to chronic blood loss from parasite feeding activity and lesions in the intestinal mucosa [3]. In 2010, hookworm infection was ranked as one of the top four causes of global anemia for both men and women [8], and was estimated to account for up to 54% of moderate-to-severe anemia in pregnant women in Africa and Asia [4].

Costs associated with the implementation of PC vary primarily by the type of delivery strategy used. Much attention has been paid to SAC and pre-SAC through pharmaceutical drug donations, large-scale national deworming programs, and global reporting systems. While WRA may indirectly benefit from PC targeting children living in the same household, direct benefits of treatment are lost, as well as additional indirect benefits accruing to the household. Despite evidence of health benefits associated with pre-pregnancy deworming and iron supplementation [9], a major barrier to including WRA in deworming programs is likely the fear of inadvertently administering deworming drugs to women who may not be aware that they are in their first trimester of pregnancy (at which time deworming in contraindicated) [10]. A comprehensive approach to PC programs targeting WRA is currently lacking.

## Subgroups of WRA

In 1994, WHO first discussed the inclusion of WRA (including pregnant and lactating women) in deworming recommendations, and called on the research community to address critical research gaps [7]. The reproductive life course of women consists of several dynamic physiological stages, each presenting unique opportunities for large-scale deworming programs (Table 1).

**Table 1:** Summary of benefits and challenges of including subgroups of WRA in deworming programs.

Subgroup	Benefits of deworming	Challenges of deworming
**Adolescent girls**	reduced partitioning of nutrients between STHs and growth during puberty easily reachable in school-based programs	fear of inadvertent administration in first trimester of pregnancy difficult to reach nonenrolled school children
**Nonpregnant, nonlactating women**	improved work performance improved nutritional status prior to conception	not easily reachable fear of inadvertent administration in first trimester of pregnancy
**Pregnant women**	of benefit to both mother and fetus easily integrated into antenatal care easily reachable in health centres and hospitals no fear of inadvertent administration in first trimester of pregnancy	some governments are hesitant to administer drugs to pregnant women despite evidence of lack of harm antenatal care attendance is low in many countries
**Lactating women**	of benefit to both mother and infant easily integrated into postpartum care easily reachable in health centres and hospitals no fear of inadvertent administration in first trimester of pregnancy if given soon after delivery	some governments are hesitant to administer drugs to lactating women despite evidence of lack of harm the number of deliveries in health care settings may be suboptimal

STHs = Soil-transmitted helminths (roundworms, whipworms, and hookworms)

### Adolescent girls

Numerous experimental and observational studies of the effectiveness of deworming on a multitude of health outcomes have been conducted in children and have included the subgroup of adolescent girls. However, few studies have been specifically conducted in this subgroup, have disaggregated data, or have reported results separately for adolescent girls. A recent Cochrane review included 45 randomized controlled trials in children aged ≤ 16 years [11]. Among trials of children known to be infected (*n* = 8), single-dose deworming was found to have had positive effects on weight, height, and mid-upper arm circumference, but not on hemoglobin (Hb) concentration. Among trials treating both infected and uninfected children in endemic areas (*n* = 37), single-dose deworming did not have an effect on anthropometry or Hb concentration. Many of the trials included in this review had short follow-up times that may have been insufficient to observe accrued benefits and were insufficiently powered to detect differences between groups due to effect dilution (i.e., including both infected and uninfected children in the treated study population). This review is also limited by the wide age range, lack of stratification by sex, and a high degree of heterogeneity among the studies. Generalizability of the review’s results to adolescent girls is therefore inappropriate.

Adolescent girls are often included in school-based deworming programs, which have been shown to be operationally feasible and highly cost-effective in a variety of settings. However, the extent to which they can benefit adolescent girls depends on school attendance and the ability to include nonenrolled children in PC activities. In many low-and-middle-income countries (LMICs), the rate of drop-out in girls during puberty spikes [12], making adolescent girls difficult to reach using school-based programs alone. Deworming programs targeting households (e.g., in those districts where all household members receive albendazole within the Global Programme to Eliminate Lymphatic Filariasis [PELF]), would include adolescent girls. Identifying other opportunities for reaching this group would arise from a local understanding of adolescent girls' social interactions, with attendant drug distribution costs related to each setting.

### Nonpregnant and nonlactating adult women

Several studies have evaluated the effectiveness of deworming in adult populations, though these have frequently included children as well, and have not adequately separated results by age group or by sex. Two studies have specifically investigated deworming in nonpregnant and nonlactating adult women. Gilgen et al. conducted a randomized controlled trial in Bangladesh among female tea pluckers and included the following four intervention groups: 1) weekly iron supplementation for 24 weeks; 2) single-dose 400 mg albendazole given at baseline and 12 weeks; 3) weekly iron supplementation for 24 weeks and single-dose 400 mg albendazole given at baseline and 12 weeks; and 4) placebos for both iron supplementation and albendazole [13]. No statistically significant difference in labour productivity was found between the intervention groups over a 20-week period. Among women who were randomized to receive deworming only, there was no statistically significant change in Hb values pre- and postintervention. Important information about the trial methods is not mentioned (i.e., blinding, attrition rate, power, and sample size calculations), and as such, its quality cannot be determined. The second study was a population-based study conducted in Vietnam. Nonpregnant women were given weekly iron-folic acid supplementation plus single-dose albendazole at four-month intervals for one year and biannual deworming thereafter over 54 months [14]. The prevalence of anemia declined from 38% to 18%, and the prevalence of hookworm infection fell from 76% to 11%. The authors did not determine the effect of deworming alone on the prevalence of anemia, nor had they included a control group for comparison.

Nonpregnant and nonlactating women are not easily reachable through health facility-based platforms. Mass drug administration of whole communities, as is done within PELFs, would provide additional coverage. Other opportunities to reach this group of women would include local settings where women might congregate, such as in marketplaces, in schools, in churches, or in other culturally-specific places, again with attendant drug distribution costs.

### Pregnant women

To date, five trials (totaling 4,265 participants) on deworming with single-dose deworming drugs during pregnancy have been conducted and are summarized in a recent Cochrane review [15]. Results indicated that deworming during pregnancy had no overall effect on maternal anemia, low birthweight, preterm birth, or perinatal mortality. This review, and the trials it includes, has several limitations. Foremost among these is that no single trial used the same intervention, and variability—in terms of sample sizes—was high. Heterogeneity in baseline prevalences and intensities of STH infections (including species-specific prevalences and intensities), also limits the appropriateness of pooling individual trial results. Additionally, treatment effects were not stratified by baseline STH intensity categories, and thus effect dilution contributes to the null findings. Infant growth outcomes were only measured at birth, or shortly after birth, which also likely limited the potential to detect a benefit. Evidence to date suggests that deworming after the first trimester of pregnancy is not associated with adverse birth outcomes [16].

Pregnant women can be reached in hospitals and health centres, where PC can piggyback on existing antenatal care services. However, ministries of health in endemic areas may be reluctant to integrate deworming into antenatal care following the first trimester of pregnancy due to fears of harming the growing fetus. This may contribute to the reason why pregnant women remain excluded from PC activities.

### Lactating women

Despite the 1994 call from WHO for research on lactation performance following deworming in lactating women [7], to date, no study has assessed the effects of deworming following delivery (i.e., in the postpartum period) on maternal or infant outcomes. While data from other WRA subgroups provide important empirical evidence, given the unique interface between mother and child during the postpartum period, they may not be applicable to lactating women. A single pharmacokinetic study of albendazole in human breast milk [17] estimated that breastfed infants of mothers who were administered a single 400 mg oral dose of albendazole were exposed to less than 0.1 mg/kg (of infant weight) of albendazole and its active metabolite, albendazole sulphoxide, over 36 hours. The authors concluded that these low concentrations would be unlikely to be considered harmful to breastfed infants.

Lactating women can be reached at multiple time points during early, mid, and late lactation. During the early postpartum period, and up to six weeks postdelivery, deworming can be effectively integrated into routine in-hospital or home visit postpartum services. They can also piggy-back on well-baby clinic visits and childhood vaccination time points [18].

## Maternal Postpartum Deworming: A New Strategy

**Fig 1:**
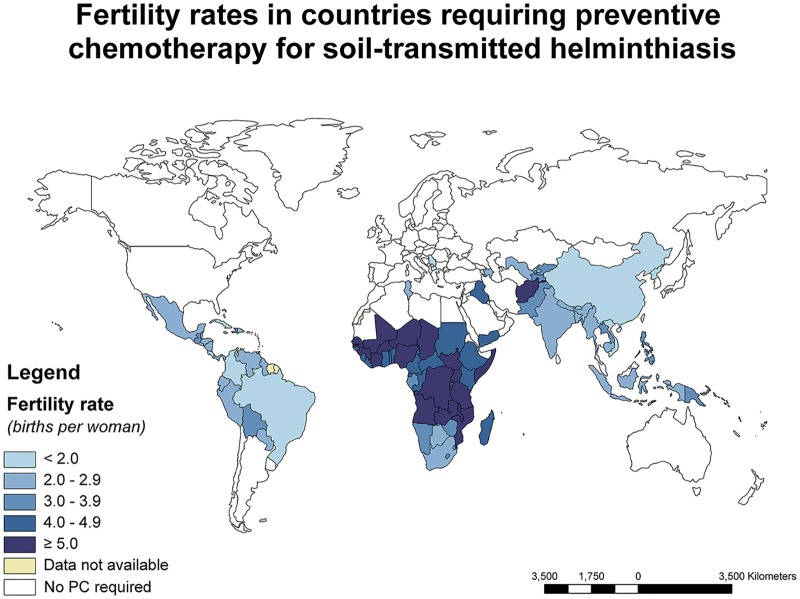
World map showing fertility rates (blue shading) among countries determined to be in need of PC for STHs according to WHO guidelines: Fertility data are taken from the World Development Indicators Databank (World Bank, 2013) and the World Statistics Pocketbook (United Nations Statistics Division, 2010–2015). Data on the need for PC in children for STH infection are taken from the WHO Preventive Chemotherapy Databank (World Health Organization, 2013). Fertility rates (in parentheses) for endemic regions that could not be displayed are: Cape Verde (2.3), Comoros (4.6), French Polynesia (2.1), Kiribati (3.8), Marshall Islands (4.1), Mauritius (1.4), Micronesia (3.3), Nauru (4.3), Sao Tome and Principe (4.6), Tonga (3.8), and Tuvalu (3.2).

Postpartum anemia is thought to affect up to 80% of women in LMICs [19]. On average, a woman in sub-Saharan Africa spends as much as 28% of her lifespan pregnant, and 65% breastfeeding [7]. A critical research gap exists on the effects of maternal postpartum deworming on infant and maternal health outcomes, including on lactation performance. The integration of deworming into routine postpartum care may prove to be an operationally feasible strategy for the inclusion of WRA in deworming efforts, since women in the postpartum period are generally easily reachable; costs associated with drug distribution can be reduced; and government policies for hospital-based deliveries are promoted. In areas of STH endemicity, fertility rates are high, offering periodic opportunities to reduce the STH-attributable burden of disease in WRA during their most reproductive years (Fig 1).

## Future Research Directions

As new plans are being discussed to include WRA in deworming activities, the most appropriate platforms, strategies, and target groups need to be considered. Comparative costing of the different strategies will be essential for program managers. In particular, as a relatively easily implementable and immediate strategy, there is a need to study the effectiveness of maternal postpartum deworming as a means to improve both maternal and child health. Deworming has the potential to improve maternal appetite, dietary intake, and nutrient absorption, and, in turn, may improve production of breast milk. Improvements to maternal nutritional status may have lasting consequences on maternal fatigue, mother–child interactions and breastfeeding, and may, overall, enable mothers to better care for their newborn infants. Research on the benefits of maternal postpartum deworming is urgently needed to build on the deworming framework that has already been developed for children, and to take advantage of the momentum generated by deworming efforts globally.
